# Linking Perception, Cognition, and Action: Psychophysical Observations and Neural Network Modelling

**DOI:** 10.1371/journal.pone.0102553

**Published:** 2014-07-16

**Authors:** Juan Carlos Méndez, Oswaldo Pérez, Luis Prado, Hugo Merchant

**Affiliations:** Departamento de Neurobiología Conductual y Cognitiva, Instituto de Neurobiología, UNAM, Campus Juriquilla, Querétaro, México; Centre de Neuroscience Cognitive, France

## Abstract

It has been argued that perception, decision making, and movement planning are in reality tightly interwoven brain processes. However, how they are implemented in neural circuits is still a matter of debate. We tested human subjects in a temporal categorization task in which intervals had to be categorized as short or long. Subjects communicated their decision by moving a cursor into one of two possible targets, which appeared separated by different angles from trial to trial. Even though there was a 1 second-long delay between interval presentation and decision communication, categorization difficulty affected subjects’ performance, reaction (RT) and movement time (MT). In addition, reaction and movement times were also influenced by the distance between the targets. This implies that not only perceptual, but also movement-related considerations were incorporated into the decision process. Therefore, we searched for a model that could use categorization difficulty and target separation to describe subjects’ performance, RT, and MT. We developed a network consisting of two mutually inhibiting neural populations, each tuned to one of the possible categories and composed of an accumulation and a memory node. This network sequentially acquired interval information, maintained it in working memory and was then attracted to one of two possible states, corresponding to a categorical decision. It faithfully replicated subjects’ RT and MT as a function of categorization difficulty and target distance; it also replicated performance as a function of categorization difficulty. Furthermore, this model was used to make new predictions about the effect of untested durations, target distances and delay durations. To our knowledge, this is the first biologically plausible model that has been proposed to account for decision making and communication by integrating both sensory and motor planning information.

## Introduction

When we need to arrive quickly to our work and we can choose between two alternative routes, we rarely compare their specific lengths. Rather, we categorize them as short or long routes. Through categorization we mentally assign environmental stimuli to groups whose members are treated similarly [1]–[5]. Thus, categorization stands out as a process linking perception with decision making and action selection. How is this implemented in the activity of neurons? Among all stimuli living beings must categorize for successful behavior, time stands out as one of the most essential [6], [7] and it has been shown that it can be encoded in the increasing or decreasing ramping activity of cell populations in different cortical areas [8]–[11]. Theoretically, based on these activation profiles, subjects could decide to which duration category an interval belongs and express this decision through a motor act.

An issue that naturally follows is the relationship between categorization difficulty and movement execution. Is there any difference in a movement when the interval that elicits it is harder or easier to categorize? Are movements always started once categorization is finished? It has been argued that categorical decisions may be arrived at either before or after the onset of the movement that is made as a consequence of the decision [12]. Thus, subjects might follow two strategies: they might delay movement onset until they are certain of their categorical decision or, alternatively, they might begin a movement with a trajectory in between both targets and decide while moving, modifying their trajectory towards the selected target [13]. In turn, reaction time (RT) and movement time (MT) would change accordingly, informing about the strategy followed by the subjects.

There are, however, other factors that can impact on these and other kinematic parameters independently of the categorization process. For example, the distance between potential reaching targets has a direct influence on RT, with slower movement onsets when potential targets appear far from each other [14], [15]. In turn, MT increases with greater movement distances and smaller target sizes [16], [17] and varies with movement direction [18]. Movement direction also modifies the trajectories and their acceleration-deceleration profile: reaching movements made with a joystick tend to be curved away from the body when performed in the lateral plane and have abrupt speed changes, whereas movements in the anterior-posterior axis tend to be straighter and have a more uniform speed [17], [19]–[21].

Although perceptual, cognitive and motor planning processes as those described above are now thought to be implemented in parallel by overlapped brain mechanisms [22], [23], how this is achieved has still to be described. Here we report the performance of human subjects on a task that involved measuring the duration of an interval, waiting 1 s for two reaching targets to appear, and communicating a categorical decision about the interval by introducing a cursor inside one of the two targets. Categorization difficulty, the distance between the targets and movement direction were varied independently to look for interactions between these variables and the categorization performance, RT and MT. Our psychophysical results were then used to look for a model that replicated these critical behavioral data. We describe a mutual inhibition network model that sequentially represented stimulus quantification, working memory, decision making, and response selection in its state without changing its connectivity. It accurately reproduced subjects’ RT, MT, and categorization performance. Moreover, it makes some testable predictions about the effect of different intervals, distances between targets and delays. Finally, this model has some strong implications about the nature of information held in working memory, the moment in which decisions are reached and how they are transformed into a movement, which could be confirmed neurophysiologically.

## Results

### Behavioral results

**Figure 1:**
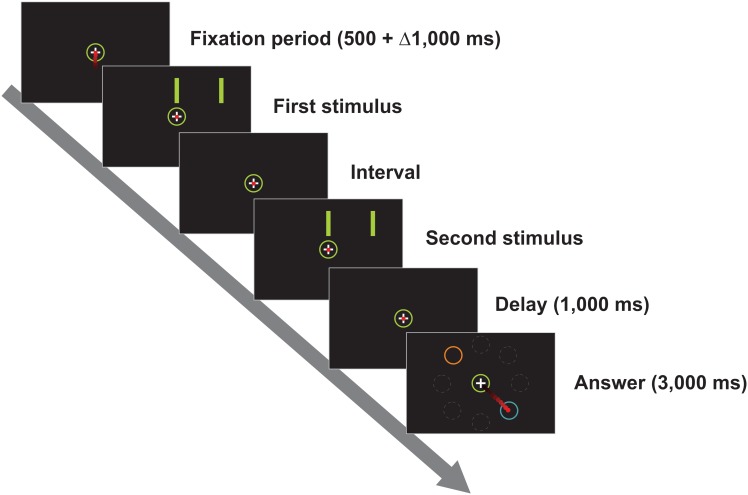
Sequence of events in a trial of the temporal categorization task. Subjects had to judge if the interval between the first and second stimulus belonged to the ‘short’ or ‘long’ category. They reported their decision by introducing the cursor inside the circle with an orange outline if their decision was ‘short’ and inside the circle with a blue outline if their decision was ‘long’. Depicted in the last frame are the eight possible positions that the response circles could adopt around the central circle. However, in a particular trial only the two response circles were visible.

Twenty human subjects completed three sessions of a temporal categorization task in which intervals had to be assigned to a ‘short’ or ‘long’ category according to previously acquired prototypes, as has been described in a previous report [24]. The intervals were between 450 and 920 ms, with intervals near the implicit central value being harder to categorize. Manipulating a joystick, subjects communicated their decision by moving the cursor inside a response circle representing their chosen category (Fig. 1). Our aim was to study the influence that the duration of the categorized interval had on the planning and execution of this movement by measuring the Reaction Time (RT) and the Movement Time (MT). To further understand MT variations we also analyzed the movement’s Maximum Velocity, the Area of its trajectory, the Number of Submovements and Decision Changes (see Methods for parameter definitions). Since response circles could appear in one of eight different positions around the central circle, we also looked for effects of the Movement Direction and the Angle of separation between these targets as these variables have been reported to influence some of our studied variables [14]–[21].

### Categorization performance

**Figure 2:**
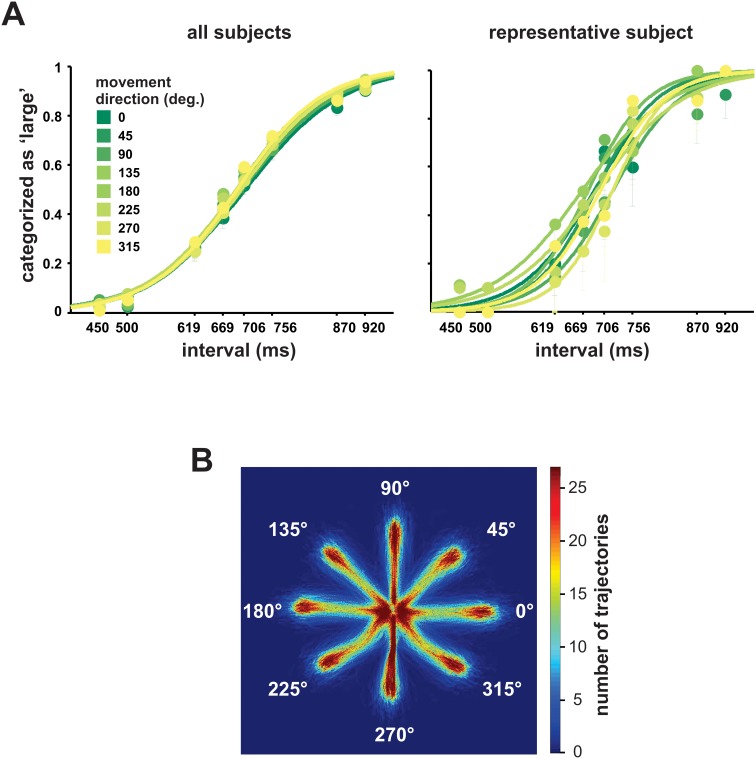
Psychometric performance of the subjects. **A)** Psychometric curves showing subjects’ performance on all movement directions. It is evident that movement direction had no influence on the final categorical decision. **B)** Number of trajectories of all the subjects that went through each pixel in the screen. Trajectories in the antero-posterior direction (90 and 270°) were straighter than other trajectories.

As expected, performance, measured as percentage of categorization errors per interval, was only affected by the magnitude of the interval being categorized as assessed with a repeated measures ANOVA with error percentage as dependent variable and the categorized interval as factor (F(7,133) = 34.11, p<0.001). Intervals near the implicit central value had more errors (Fig. 2A). On the contrary, performance was not influenced by either the Movement Direction (F(7,133) = 0.662, p = 0.637) nor the Angle between the response circles (F(2,38) = 0.116, p = 0.885). We also used the psychophysical difference threshold (Movement Direction F(7,133) = 1.603, p = 0.167; Angle F(2,38) = 0.595, p = 0.548) and the constant error (Movement Direction F(7,133) = 1.91 p = 0.106; Angle F(2,38) = 1.611, p = 0.216) as dependent variables and the Movement Direction and Angle between targets as factors with equal results. This implies that neither the direction of the movement nor the angle between the possible targets modified subjects’ categorical outcome (see Fig. 2A).

### Stimulus magnitude

**Figure 3:**
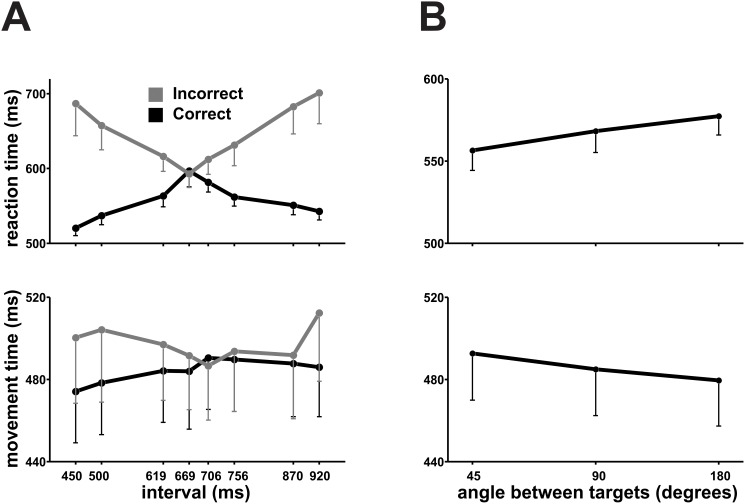
Reaction and movement times. **A)** Mean RT and MT as a function of the categorized interval, with trials subdivided according to their outcome. **B)** Mean RT and MT as a function of the angle between targets. Errorbars = 1 SEM.

To study the effect of categorization difficulty on our execution variables we carried out a repeated-measures ANOVA with the categorized Interval as factor (see Dataset S1). All key parameters showed significant effects for Interval, namely, RT (F(1,133) = 19.2, p<0.001), MT (F(1,133) = 3.68, p = 0.005), and Number of Submovements (F(1,133) = 2.73, p = 0.017), but not Decision Changes (F(1,133) = 0.85, p = 0.48). This suggests that categorization difficulty does not only affect the decision process and the movement planning (RT), but also its execution (MT and Submovements), with larger RTs and MTs for the intermediate intervals that were harder to categorize (Figure 3A). An additional two-way ANOVA with Interval and Outcome (correct or incorrect categorizations) as factors, showed a significant main effect of Outcome for RT (F(1,11) = 29.423, p<0.001), MT (F(1,11) = 5.761, p = 0.035), Number of Submovements (F(1,11) = 5.717, p = 0.036), and Decision Changes (F(1,11) = 10.241, p = 0.008), as well as an Interval×Outcome interaction effect for RT (F(7,77) = 5.142, p = 0.002). In fact, subjects showed larger RTs and MTs for incorrect trials on the extreme intervals (Figure 3A) which were the easier to categorize, emphasizing their ability to detect an error and, in some cases, correct their categorical decision. Overall, these findings suggest that even after a delay period of a second, the Interval and the categorical Outcome had a large effect on RT and a smaller effect on the MT profile, suggesting that the decision making and its translation to a motor command were continuously being revised during these epochs.

### Angle between targets

**Figure 4:**
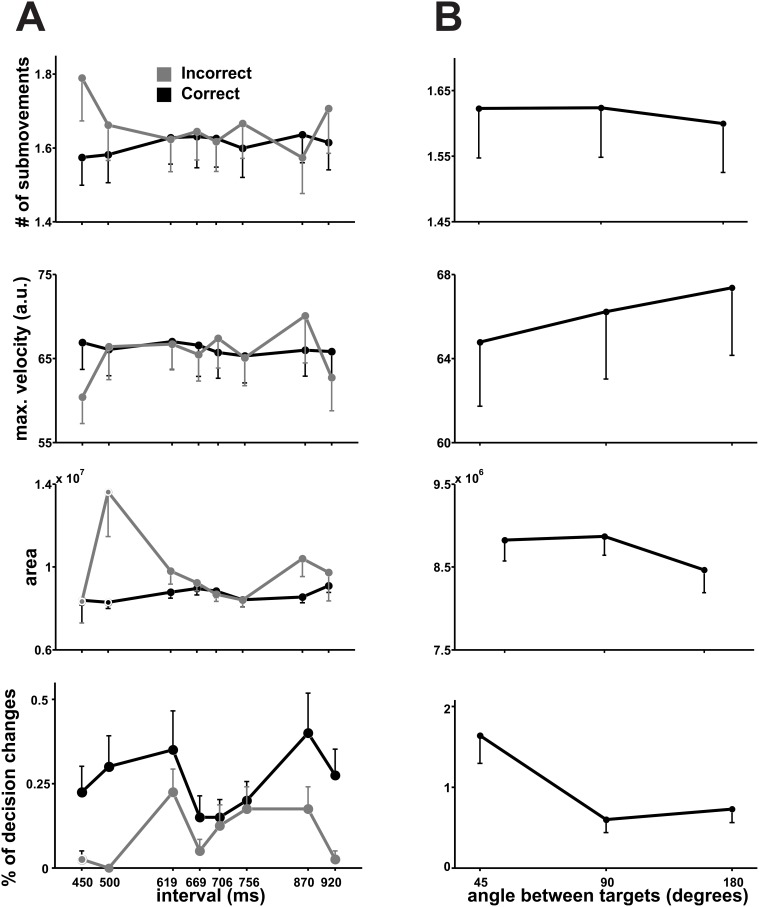
Mean number of submovements, maximum velocity, area of the trajectories and percentage of Decision changes as a function of interval duration (A) and angle between the targets (B). Errorbars = 1 SEM.

The angle subtended by the targets had a profound influence on movement preparation and execution parameters (see Dataset S2), except the Number of Submovements (RT F(2,38) = 11.99, p<0.001; MT F(2,38) = 10.738, p<0.001; Area F(2,38) = 3.4, p = 0.047; Maximum Velocity F(2,38) = 14.017, p<0.001; Decision Changes F(2,38) = 9.687, p = 0.002). As shown in Figure 3B, it is evident that RT increased as a function of this angle, whereas MT decreased. The Area, Decision Changes, Number of Submovements and Maximum Velocity (Fig. 4B) evidently varied in accordance with MT’s changes, as has been reported in other studies [25], [26]. This pattern can be due to subjects beginning their movement before they reach a decision (shorter RT) and deciding while they move (longer MT) as long as targets are close to each other, since in this case a slight trajectory modification would be enough to reach the desired target. Another explanation may be that contiguous targets elicit a quick, although gross, representation of movements in their direction in motor circuits (short RT) which are then refined for precision (long MT). Of course, these two explanations are not mutually exclusive.

### Movement Direction

**Figure 5:**
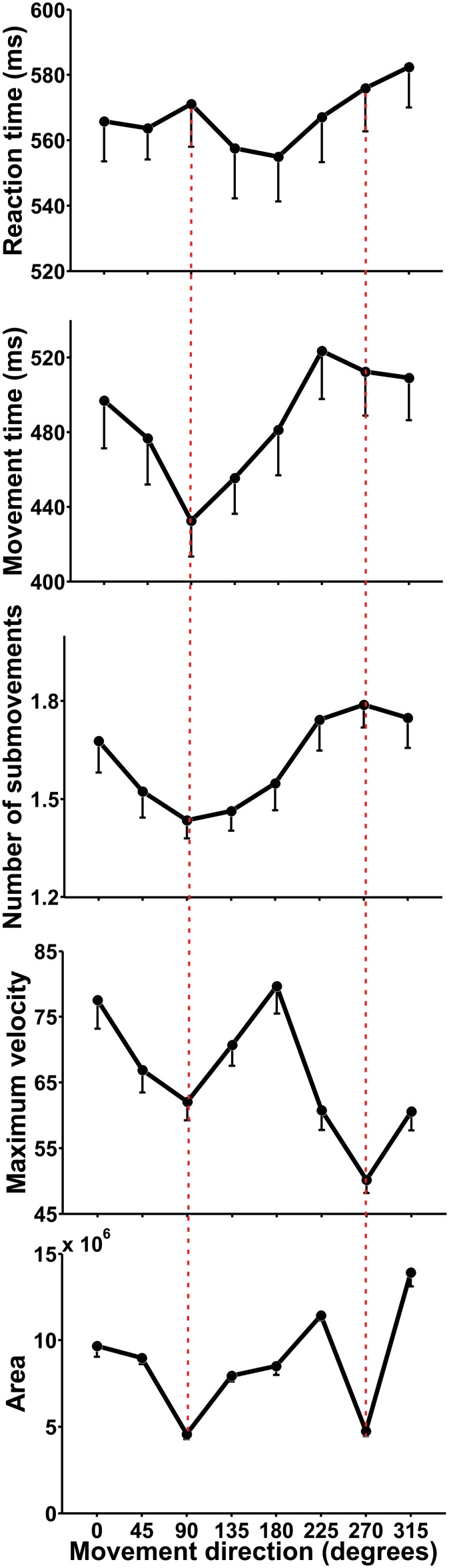
Mean RT, MT, number of submovements, maximum velocity and area of the trajectories as a function of movement direction. Errorbars = 1 SEM.

Even though movement direction had no influence on categorization performance, it influenced all of our variables except the occurrence of Decision Changes (RT F(7,133) = 3.043, p = 0.017; MT F(7,133) = 21.861, p<0.001; Number of Submovements F(7,133) = 19.292, p<0.001; Area F(7,133) = 51.528, p<0.001, Maximum Velocity F(7,133) = 71.963, p<0.001). A closer inspection reveals a complex pattern, where the Area of the trajectories was smaller for movements in the anterior-posterior axis (90 and 270°; Fig. 2B), Maximum Velocity was greatest for medio-lateral movements (0 and 180°) and more Submovements were made in the posterior direction (270°). Although MT was also clearly influenced by movement direction, none of the aforementioned kinematic variables seems to solely explain its profile and rather a combination of them may shape MT (Fig. 5). Similar findings have been reported previously [27]–[30]; nevertheless, it is important to emphasize that the effect of movement direction on kinematics is independent from the effect of the perceptual and categorization processes that elicit the movement described above. This is supported by the lack of significant interactions between Movement Direction and Interval on any of our variables as assessed with a two-ways ANOVA (p>0.1). Furthermore, a Movement Direction x Angle between targets analysis only revealed a significant effect on the number of Decision Changes (F(14,266) = 2.64, p = 0.035).

### Modeling

**Figure 6:**
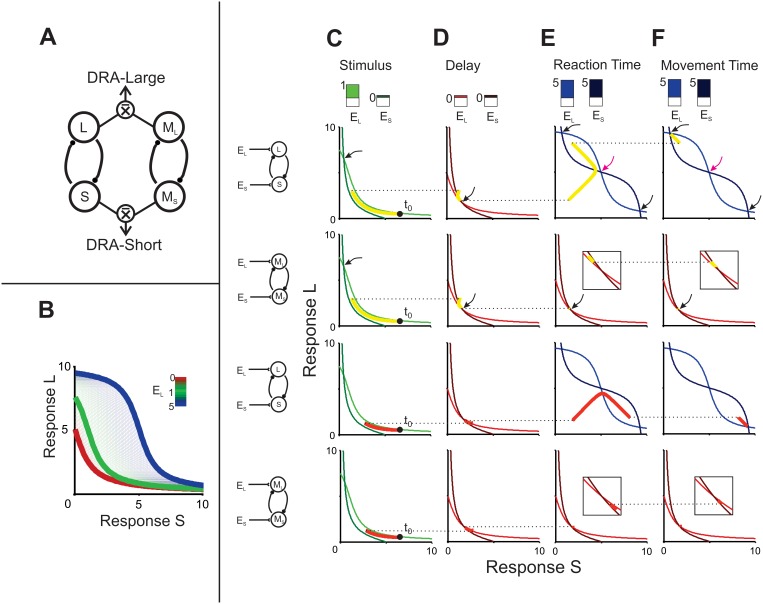
Mutual inhibition model for categorical decisions. **A)** A coupled system model composed by two groups of two mutually inhibited nodes, where L and M_L_ are tuned to large intervals and S and M_S_ for short intervals. **B)** L nullcline as a function of response of S for different values of E_L_ (inset colorbar). Thick lines correspond to values used in stimulus, delay, RT, and MT phases. For the M_L-_ M_S_ network the same nullclines were used except for the blue one, associated with the RT and MT dynamics. **C–F)** Stimulus, delay, RT, and MT phases respectively with their corresponding L and S (first and third rows) or M_L_ and M_S_ (second and fourth rows) nullclines. For each figure, top panel shows input values EL and ES as shown in B inset. Main panels show a single path for 450 ms (first row L–S; second row M_L-_ M_S_) and 920 ms (third row L-S; forth row M_L-_ M_S_) intervals. Horizontal dotted lines specify the state of the network at the end of a task phase (left) and the beginning of the next phase (right). The filled circle indicates the initial start of the network (t_0_). The black arrows indicate the point of intersection between the L and S (or M_L_ and M_S_) trajectories, which correspond to the sink in the dynamics of the mutually inhibited pairs. The arrows in magenta indicate the source associated with the repelling state of the network. The parameters for the sigmoid function were M = 10 and 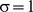

**Figure 7:**
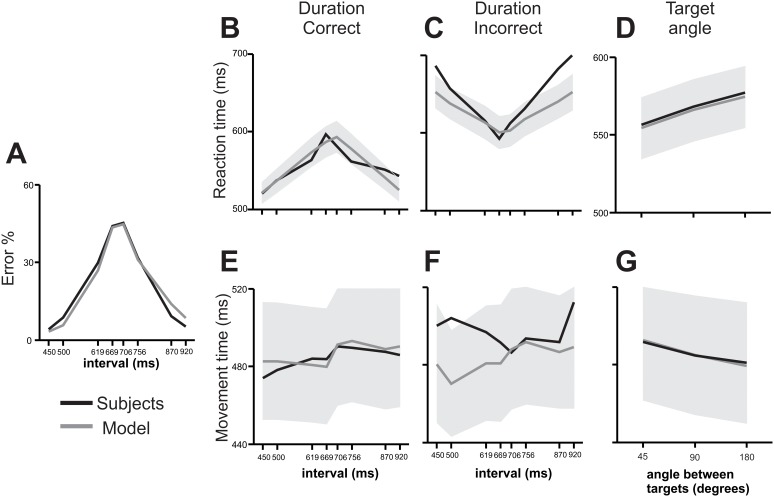
Performance of the subjects and of the model without the memory and downstream reading units. (**A**) Performance of the subjects (black line) and of the model without the memory and downstream reading units (gray line) as a function of categorized interval. (**B**) RT of the subjects (black line) and model (mean, gray line; ±0.12SD gray area) as a function of interval for correct trials. (**C**) RT of the subjects (black line) and model (mean, gray line; ±0.12 SD gray area) as a function of interval for incorrect trials. (**D**) RT of subjects (black line) and model (mean, gray line; ±0.12SD gray area) as a function of the angle between targets. (**E**) MT of the subjects (black line) and model (mean, gray line; ±0.12 SD gray area) as a function of interval for correct trials. (**F**) MT of the subjects (black line) and model (mean, gray line; ±0.12SD gray area) as a function of interval for incorrect trials. (**G**) RT of subjects (black line) and model (mean, gray line; ±0.12 SD gray area) as a function of de angle between targets.

**Figure 8:**
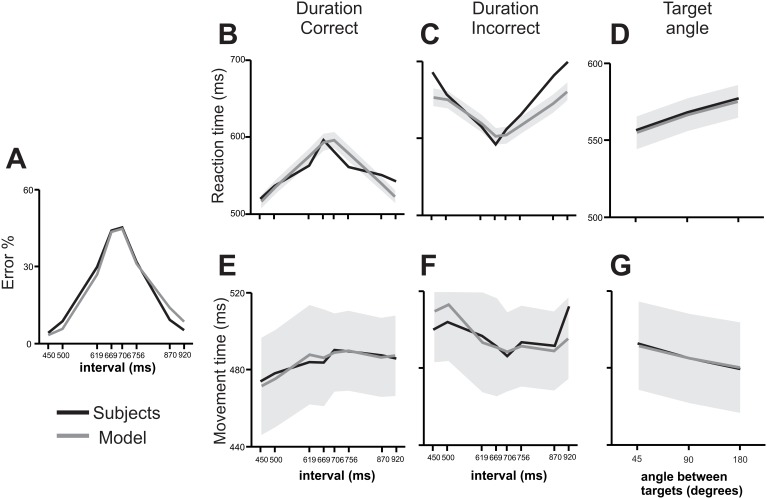
Performance of the subjects and of the model with the memory and downstream reading units. (**A**) Performance of the subjects (black line) and of the complete model (gray line) as a function of categorized interval. (**B**) RT of the subjects (black line) and model (mean, gray line; ±0.12SD gray area) as a function of interval for correct trials. (**C**) RT of the subjects (black line) and model (mean, gray line; ±0.12SD gray area) as a function of interval for incorrect trials. (**D**) RT of subjects (black line) and model (mean, gray line; ±0.12SD gray area) as a function of the angle between targets. (**E**) MT of the subjects (black line) and model (mean, gray line; ±0.12SD gray area) as a function of interval for correct trials. (**F**) MT of the subjects (black line) and model (mean, gray line; ±0.12SD gray area) as a function of interval for incorrect trials. (**G**) RT of subjects (black line) and model (mean, gray line; ±0.12SD gray area) as a function of de angle between targets.

Overall, the previous results suggest that categorization and decision-making processes are mixed-up with movement planning, where time quantification, its assignment to a category, and the evaluation of potential movements reverberate in the brain before and during decision communication. We developed a neural network model that aimed to replicate our results on key behavioral parameters, namely subjects’ categorical performance, RT and MT as a function of stimulus duration and outcome, as well as the angle between the targets. This model included four phases: 1) an information accumulation phase delimited by the time elapsed between the two stimuli, 2) a delay in which stimuli were stored in working memory, 3) a reaction time phase in which the targets were presented and the movement that expressed the categorical decision was prepared, and 4) a movement time phase for the execution of the reaching movement to one of the targets. Inspired by Machens et. al. [31], it implied the interaction between two mutually inhibiting neural populations shaped to prefer one of two categories, short (*S*) or long (*L*; Fig. 6A). These nodes were modulated by independent excitatory inputs that varied depending on the phase of the task, thus varying the network’s state, which in turn represented time passage, temporary memory storage, decision making, and movement execution (Fig. 6B–F). This network configuration was able to replicate the categorical performance, the effect of interval duration on RT for correct and incorrect trials, as well as the RT and MT changes as a function of the angle between targets (Fig. 7A–E), but could not replicate the effects of interval duration on MT, particularly for incorrect trials (Fig. 7F, Table 1). However, when we added another pair of mutually inhibiting neural populations (*M_L_* and *M_S_*) that kept in working memory the interval information, the model accurately accounted for all of the aforementioned variables (Fig. 8, Table 1).

**Table 1:** Linear regression fittings for reaction time (RT) and movement time (MT) as a function of interval duration, with and without the addition of the memory (M) and downstream reading (DRA) nodes.

	With M and DRA nodes		Without M and DRA	
	F	p	F	p
RT Correct	37.43	0.00	29.70	0.00
RT Incorrect	61.70	0.00	129.77	0.00
MT Correct	50.29	0.00	5.82	0.05
MT Incorrect	3.00	0.13	0.36	0.57

**Figure 9:**
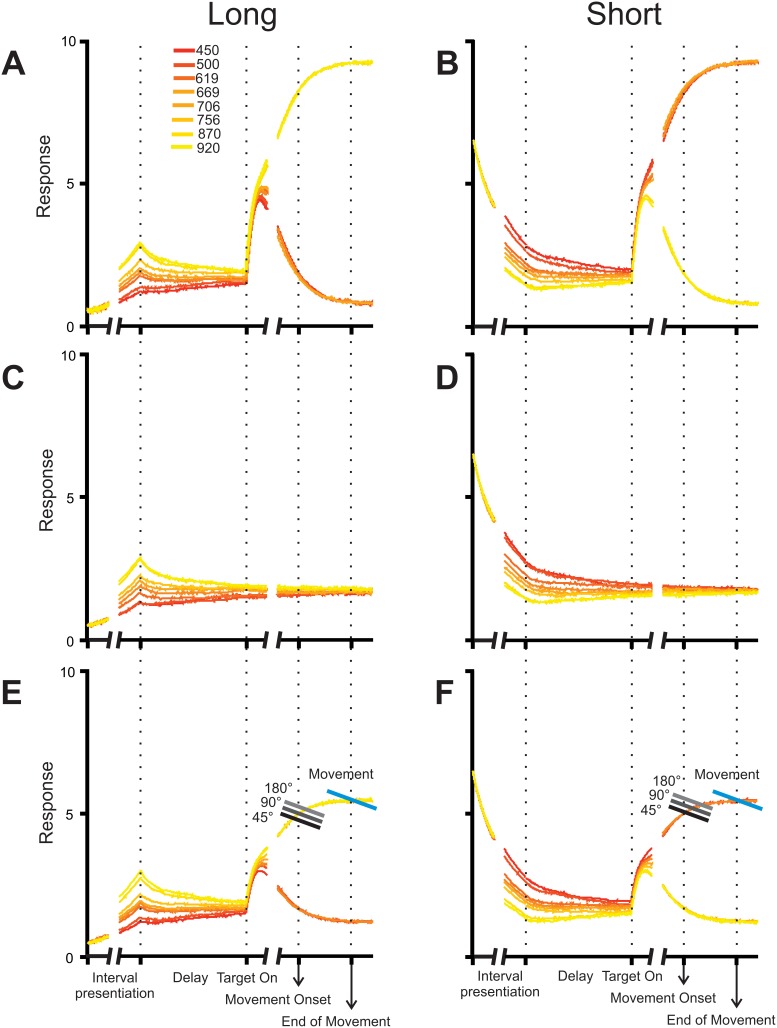
Model Trajectories as a function of task epochs. Response of L (**A**)**,** S (**B**), M_L_ (**C**)**,** M_S_ (**D**), and the downstream reading areas DRA_L_ (**E**) and DRA_S_ (**F**) to the intervals used in out categorization task. In E and F the trajectories were classified as long or short if they reached an initial threshold that depended on the angle of separation between the targets (gray scale). MT is defined as the period that it takes for the trajectory to move from the first (gray) to the second threshold (blue line). Trajectories are aligned to the beginning of the delay on the left side and to movement onset on the right side.

During the interval presentation phase, the activity of *L* and *M_L_* increased whereas that of *S* and *M_S_* decreased as a function of interval duration, reaching a particular level at the end of the phase (Fig. 6C), simulating the increasing and decreasing ramping activity previously described in neurophysiological experiments [8]–[11]. This level was maintained during the delay; however, there was a decay in information as this phase advanced, showing a tendency for the activity in all cases to reach the mean, as has been observed empirically [32], [33] (Figs. 6D, 9A, B). If the delay period were extended, activity would ultimately reach this mean, which would represent a correlate of forgetfulness. Once the targets appeared, the answering phase began and this was observed as a sudden increase of activity in *L* or *S* nodes, while *M_L_* and *M_S_* continued the maintenance of interval information in the leaking working memory (Fig. 9C, D). Soon the activity in one of the *L* or *S* nodes dominated and inhibited the other node. Thus, the trajectory that described the network’s state was abruptly driven towards one of two attractors during this RT phase (Figs. 6E, 9A, B). Importantly, two downstream reading areas (*DRA*), one for each category, integrated the output of their corresponding nodes: *DRA_L_* computed the mean of *L* and *M_L_*’s output, whereas *DRA_S_* did the same for *S* and *M_S_* (Fig. 6A, 9E, F). These areas were also driven towards their corresponding attractor and when one of them crossed a threshold, it triggered a movement towards the target that represented its preferred category (Fig. 6E). This threshold differed depending on the angle subtended between the targets: thus, the narrower the angle, the shorter the RT (Fig. 9E, F). Finally, the period between the crossing of this threshold and the moment at which the downstream area trajectory passed through a second and final threshold (that was close to the attractor) was defined as the MT (Fig. 9E, F).

In Figure 8A, the percentage of incorrect decisions of the human subjects (black) and the complete model simulations (gray) is plotted as a function of interval duration. It is evident that the model explains almost perfectly the categorical performance of human subjects (chi^2^-test (7) = 0.15, p<0.001). In addition, the mean RT of the subjects is plotted together with the model’s RT mean ± SD across interval durations (Figure 8B correct and Figure 8C incorrect trials) or for the three possible target angles (Figure 8D). Again, the model can explain both the increase in RT for the intermediate durations that are difficult to categorize, as well as the linear increase in RT as a function of the target angle. This latter observation suggests that the movement planning to targets in opposite directions takes a larger time, as reported previously [14], [15], [46]–[50]. Indeed, according to chi^2^-tests comparing the RT distribution of the model with that of the subjects, the model properly explained RT across correct categorization trials (correct durations chi^2^-test (7) = 2.3, p<0.05; but not incorrect durations chi^2^-test (7) = 6.1, p = 0.4) and across target angles (chi^2^-test (2) = 0.02, p<0.001). Furthermore, we carried out regression analyses between the predicted values of the model and the actual subjects’ data, which showed that both correct and incorrect trials’ RT was accounted for by the model (Table 1) and the same was true for the regressions made with the angle between the targets (Table 2). Also, the MT observed in the model simulations follows the concave shape of subjects’ MT as a function of interval duration for correct trials (Figure 8E) and the convex shape for incorrect trials (Figure 8F), and perfectly overlaps with the humans’ decrease in MT as a function of target angle (chi^2^-test (2) = 0.01, p<0.001; Figure 8G, Table 2), as well as for the correct (chi^2^-test (7) = 0.09, p<0.001, Table 1) and incorrect interval categorizations (chi^2^-test (7) = 0.9, p<0.001).

**Table 2:** Linear regression fittings for reaction time (RT) and movement time (MT) as a function of the angle between the targets with and without the addition of the memory (M) and downstream reading (DRA) nodes.

	With M and DRA nodes		Without M and DRA nodes	
	F	p	F	p
RT	6101.02	0.01	4524.60	0.01
MT	121260.12	0.00	242.43	0.04

**Figure 10:**
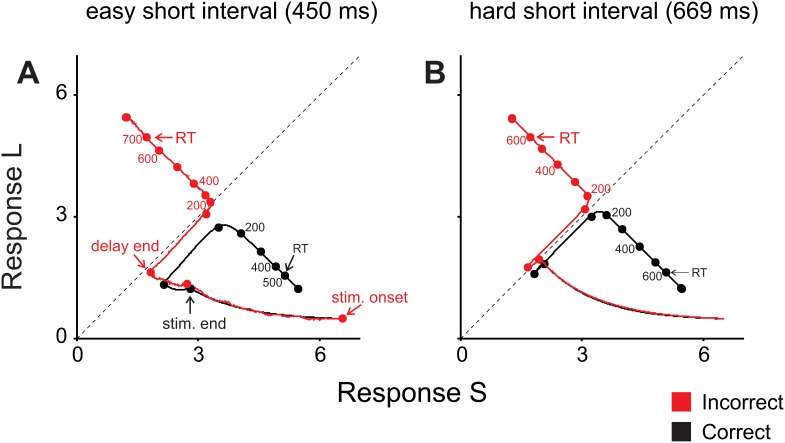
Network’s trajectories for correct and incorrect trials. Mean paths of the network’s trajectories during correct (black) and incorrect (red) categorization trials using the easiest short interval (450 ms, **A**) and the hardest short interval (669 ms, **B**). It can be appreciated that in “easy” trials (**A**), incorrect trajectories travel very close to the midline between attractors and remain there for a longer period than incorrect trajectories in “hard” trials (**B**), which are almost mirror images of the corresponding correct trajectories. This in turn explains the longer RT in incorrect “easy” trials than in “hard” ones.

Figure 10 shows in detail the network dynamics during easy and hard categorization trials and explains the differences seen in RT as a function of interval and outcome. It can be appreciated that the correct and incorrect trajectories are almost mirror images when the hard interval is categorized (Fig. 10 B), with RT happening practically at the same long time. However, when the easy interval is categorized (Fig. 10A), the incorrect trajectory spends a significant amount of time in the source (or repulsion) zone and then travels to the incorrect attractor, whereas the correct trajectory promptly deviates to the correct attractor, thus explaining the observed marked differences in RT.

**Figure 11:**
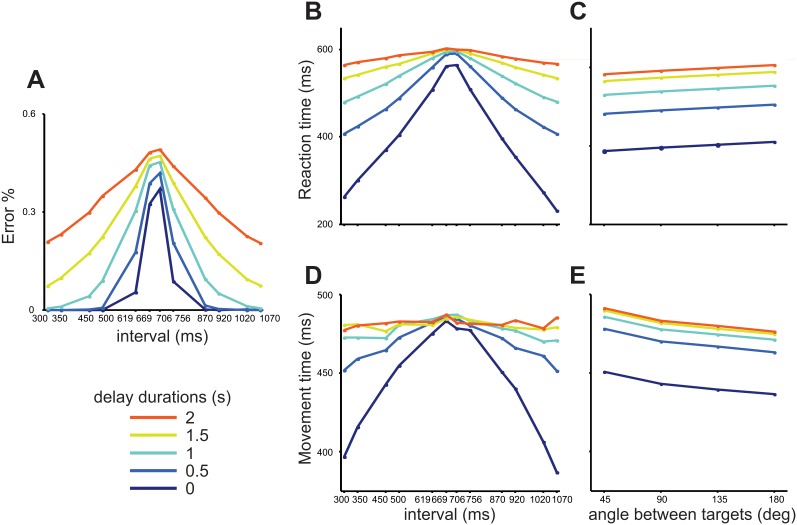
Model Predictions. Predicted performance (**A**), reaction time (**B**) and movement time (**D**) with new, untested intervals and delay durations (see bottom for color codes). Also shown are the predicted reaction (**C**) and movement (**E**) times with an untested angle (135 deg) and the untested delays.

Finally, we used our model to predict what would happen to the performance, RT and MT if we test using more intervals, one more angle between the targets, and different delay durations. As seen in Figure 11A, our model predicts that performance would become progressively worse for longer delay durations; the best performance possible would be achieved if the delay were completely removed. It can also be seen that shorter and longer intervals than the ones presented to our subjects would be categorized with fewer errors, as expected. Another interesting prediction is that both RT (Figure 11B) and MT (Figure 11D) would increase as a function of delay duration, particularly for the shorter and longer intervals, potentially reaching an asymptote in which all intervals would elicit the same RT and MT. These same parameters were predicted to keep changing as a function of the angle between the targets, as seen in Figure 11C, E in which a separation of 135 degrees, which was not tested, is depicted.

## Discussion

We tested human subjects in a temporal categorization task that varied in difficulty and in which subjects had to move a cursor with a joystick in different directions to communicate their decision. The task involved several cognitive abilities: subjects had to quantify the passage of time, decide if the interval belonged to the *short* or *long* category, and select the corresponding target (Fig. 1). Working memory was also involved since there was a 1 s delay between interval presentation and decision communication, forcing subjects to hold in memory the interval duration or the categorical decision during this period. We mimicked our main behavioral results with a model consisting of two neural populations, each composed of two nodes, tuned to a preferred category. These populations had independent excitatory inputs that modulated their activity throughout the different task epochs (Fig. 6A). Nodes accumulated temporal information in the form of ramping activity, maintained that information in working memory during the delay and then competed against each other to drive the networks’ trajectory in their direction as an attractor, thus representing a decision [34]. The model replicated subjects’ performance, as well as the RT and MT, as a function of the categorized interval, the categorization outcome, and the angle between the targets.

Numerous brain regions have been involved in the processing of temporal information, but there seems to be a predominance of the premotor and supplementary motor areas, the posterior parietal cortex, the basal ganglia, and the cerebellum [35], [36]. Moreover, neural responses related to temporal information accumulation have been recorded in some of these areas. For example, neurons in the posterior parietal cortex increased [8] or decreased [11] their firing rate as the passage of time made a saccade target in their receptive field more or less relevant, respectively. Also, Merchant et al. [10] found that some neurons in the medial premotor cortex, called time-accumulator cells, increase their firing rates in a linear fashion as a function of elapsed time after a movement was executed in a rhythmic tapping task. Thus, in our model, temporal information was gradually accumulated as an increase in the activity of one set of independent neural populations (*L, M_L_*), which in turn inhibited another one (*S, M_S_*). In this way, as the intervals became larger, *L’s- M_L_* activity increased and *S’s- M_S_* decreased. This double-coding strategy, in which the activity of two populations with opposite ramps codes the same stimulus, has been shown to increase the signal to noise ratio for perceptual decisions [37] and has been recorded in timing tasks [9], [10].

However, time sensation was not enough to solve this task: information had to be categorized before a response could be communicated. Theoretically, categorization could have taken place as soon as the interval had been delimited, in which case the information stored during the delay would have been the final decision. Importantly, RT’s would be short and very similar in all trials, since subjects would only need to transform their already-made categorical decision into a movement command towards the corresponding target. Alternatively, the decision could be postponed until a response was prompted or even after response onset, in which case the interval information would be the one maintained during the delay. However, with this strategy RT’s would vary as a function of the categorization difficulty and they would be longer, since subjects would first need to make the decision and then the appropriate sensorimotor transformations. This second option is what our psychophysical results support given that subjects’ RT and MT varied depending on stimulus categorization difficulty and trial outcome, even after the 1 s delay. Indeed, a recent paper [38] reports that in monkeys performing a vibration discrimination task in which decision communication was postponed, more cells coded for the past stimuli than for the final decision during the delay, although there is neurophysiological evidence of both kinds of responses [39]. Thus, in our model, it was the duration of the interval which was maintained throughout the delay as a relatively stable state of the two mutually inhibiting networks after information accumulation ended. This kind of parametric maintenance of information in working memory has been known for decades [40].

In fact, we also modeled the effect of untested intervals and delay durations (Figure 11) on performance, RT and MT. While the effect of easier-to-categorize intervals is quite as expected, with progressively shorter and longer intervals having fewer errors, the effect of different delays has strong implications: first of all, the best moment to take a perceptual decision in this task is as soon as the interval presentation ends. We are claiming that subjects postponed their decision until they were prompted to answer, holding interval duration in a leaking and increasingly uncertain state. Thus, the longer the subject waits, the less reliable the information he has to make a categorical decision. In hand with this is the fact that the network’s state will take longer to reach one of the two possible attractors, thus increasing RT for progressively longer delays.

Once the targets were presented, separated from each other by 45, 90 or 180 degrees, subjects had to communicate their decision by introducing the cursor inside the corresponding target. As reported previously [14]–[18], the angle between the targets had an effect on RT and MT, as well as on Decision Changes, such that the narrower the angle, the shorter the RT, the longer the MT and more Decision Changes occurred. In our model, when the targets were presented the *L-S* network state was suddenly perturbed by equal excitatory inputs to both nodes. Thus, if the previous state was closer to *L*, it was attracted to this node with a greater force than to *S* and vice versa. The downstream reading area added the output of the two mutually inhibiting neural populations (Fig. 6A), containing both the trajectory towards one of the attractor states (*L–S*) and the parametric memory trace of the interval duration (*M_L-_M_S_*) (Fig. 9). Once this area reached a RT threshold, the movement was generated. Importantly, we incorporated three different thresholds for movement onset that depended on the angle between both targets: the narrower this angle was, the lower the threshold was. This implied that, regardless of the magnitude of the stimulus, RT was smaller and MT larger for targets that lied close to each other. As shown in Figure 8, our model accurately predicted RT, MT and Decision Changes as a function of the angle between the targets. We think that this effect could be due to two options that are not mutually exclusive: a) subjects had not made a decision by the time the targets were presented and, if the distance between them was short, they began moving early without having decided (short RT) and decided while moving (long MT), given that a minor trajectory change would be enough to reach the selected target; b) a non-target lying close to a target enhances a movement representation in their direction (short RT), while a non-target far from a target represents a distractor, as has been shown behaviorally [13]–[15], [41]–[44] and neurophysiologically [45]. For example, neural activity related to movement preparation varies depending on the angular separation between potential reaching targets in monkey primary motor [46] and premotor cortex [47] as well as in the human premotor, parietal [48] and sensorimotor cortex [49], [50]. In these reports, the measured signal is observed to reach a particular value before and with greater amplitude when target separation is smaller, a feature mimicked in our model with the addition of the different movement onset thresholds that varied with target angle. Needless to say, our results can’t discriminate between these options so further experiments are needed to clarify this issue.

Finally, given that intervals that were harder to categorize lied near the implicit interval and generated a state that thus lied almost equally close to both nodes, they were more prone to be attracted to the incorrect node than easier intervals, therefore making errors more likely in these cases. As seen in Figure 8A, D, performance was also well predicted by the model. Thus, this relatively simple model instantiates our key variables and represents a plausible biological mechanism behind perception, decision and action. Although we are not claiming that it is implemented in a particular area, it does imply that processes as different as time estimation, working memory, categorization, decision making and response selection might take place in a small, albeit surely distributed, interconnected network, probably in the motor system [6], [51]–[54]. Furthermore, our critical variables, RT, MT and performance are represented by one single variable, namely, the state of this network [55]. Previous models [56], [57] have shown that both decision making and working memory could be implemented in the same circuit in various ways but, to our knowledge, none has incorporated movement related considerations into it. However, the fact that neurophysiological activity related to working memory and perceptual decisions has been recorded in the same regions where motor preparation and execution takes place makes our model a reasonable one. This also supports the addition of nodes dedicated only to keeping stimulus information in the form of working memory: as has been reported from neurophysiological experiments, some neurons maintain stimulus information during a delay before response selection [40] and even during the response time [38], in dorsolateral prefrontal cortex and medial premotor cortex, respectively. This activity occurs while, simultaneously, other neurons code for other task related variables [39]. In our model, the L–S network alone was unable to replicate the effects of interval duration on MT, but with the addition of both, another pair of mutually inhibiting neural populations (*M_L_* and *M_S_*; which kept in working memory the interval information) and of a reading downstream network, we were able to simulate the perceptual and motor performance as a function of interval, the categorization outcome, and the angle between targets (Fig. 8).

Other models of sensory information accumulation and decision making have been proposed to account for these processes [12]. In fact, we also tested another decision-making model based on information accumulation superimposed in a random walk trajectory, in which a decision was made once a noisy trajectory reached a decision boundary when biased by information (see Methods). However, even though this model was simpler, it failed to replicate our data as faithfully as the model we report here and as seen for other tasks [58]. Overall, our psychophysical results and the model we propose show that a relatively simple and biologically plausible neural circuit can, when given some preprocessed sensory data, accumulate and memorize this information, categorize it and select an appropriate course of action. In turn, its activity could be fed to motor circuits, thus linking perception with movement execution.

## Materials and Methods

### Ethics Statement

The study was approved by the National Autonomous University of Mexico Institutional Review Board. All procedures complied with the Declaration of Helsinki.

### Subjects

Twenty human volunteers (9F, 11 M; age 27.1±5.2 years (mean ± SD)) participated in this study. Subjects were verbally informed about the general procedures and gave written consent before commencement of experiments. Subjects were right-handed, had normal or corrected vision and were naive about the task and purpose of the experiment. The study was approved by the National Autonomous University of Mexico Institutional Review Board. All procedures complied with the Declaration of Helsinki.

### Apparatus

Subjects were seated comfortably facing a computer monitor (HP 7540, 170Hz refresh rate) with the chin and the forehead placed in a custom-made headrest that kept the subject’s eyes approximately 56 cm from the center of the monitor. A joystick (H000E-NO-C, CTI electronics, Stratford, CT, USA) was manipulated by the subjects with their right hand to control the position of the cursor. This joystick was fixed in a platform under the desk, which prevented the subjects from seeing their own hand.

### Task

Subjects were tested on a temporal categorization task that was designed and programmed using Visual Basic (Microsoft Visual Basic 6.0, 1998). This task has been described in detail elsewhere [24]. Briefly, subjects were required to categorize as ‘short’ or ‘long’ eight different time intervals (450, 500, 619, 669, 709, 756, 870 and 920 ms) according to a previously acquired criterion. The temporal sequence of a trial is depicted in Figure 1: Subjects were required to introduce and maintain the cursor inside a 4°-diameter central circle. After a variable delay (500+Δ1,000 ms), two parallel bars (8°×0.7° of visual angle) appeared briefly above the central circle, disappeared for a particular interval (*t*), and reappeared in the same position. Crucially, *t* changed from trial to trial and was the variable that had to be categorized. After a 1-s delay, two response circles appeared in the periphery of the central circle, one with an orange outline and the other with a blue outline. Subjects communicated their decision by moving the cursor to the orange-outlined circle if their choice was ‘short’ or to the blue-outlined circle otherwise. These response circles could occupy one of eight possible locations around the central circle and in each particular trial one particular combination of positions was randomly chosen (see Fig. 1 bottom). In fact, we restricted response-circle positions to 20 different combinations to restrict the angle subtended between these circles to three values: 45, 90 and 180 degrees with the center of the screen as the vertex. This also helped to balance movement directions (an a posteriori Chi square analysis revealed that there was no predominance of particular movement directions, p<0.001). Importantly, this task design precluded the formation of precise motor plans until the response circles appeared on the screen and also allowed us to separate kinematic and categorization-related effects.

The first 24 trials of each task were part of the “Training Phase”. In this phase, only the shortest and the longest stimuli were presented in a random fashion, generating a mental implicit value that would correspond to the stimulus midway between the two extremes and would serve as a limit or boundary between categories. The color of the stimulus bars matched the color of the correct target, helping the subject to make a color-category association. The words “correct” or “incorrect” appeared at the end of the trial as feedback. The 160 trials that followed immediately made up the “Testing Phase” in which the eight values were presented randomly from trial to trial 20 times each. Each value was presented once with each one of the 20 different response-circle position combinations, with a randomized presentation sequence. In this phase, the color of the stimulus bars was always green, regardless of the stimulus category. No feedback was given in this phase. Subjects performed three separate sessions on different days, for a total of 480 testing phase trials.

### Data analysis

Subroutines written in Matlab (MathWorks v. 7.6.0.324) were used to obtain the kinematic parameters. We first projected all the trajectories to a common direction (0 degrees) by subtracting the angle of the chosen response target to each of the trajectory samples. Then, we applied a low-pass Butterworth filter with a cutoff frequency of 20 Hz and a Kalman filter. From these projected and smoothed trajectories we obtained the movement-related parameters described below. The SPSS statistical package (version 12, SPSS, Chicago, IL) was used for the statistical analyses, in which the level of statistical significance to reject the null hypothesis was α = 0.05. The analyses were conducted only on the data of the Testing Phase.

#### Reaction Time (RT), Movement Time (MT) and Maximum Velocity

**Figure 12:**
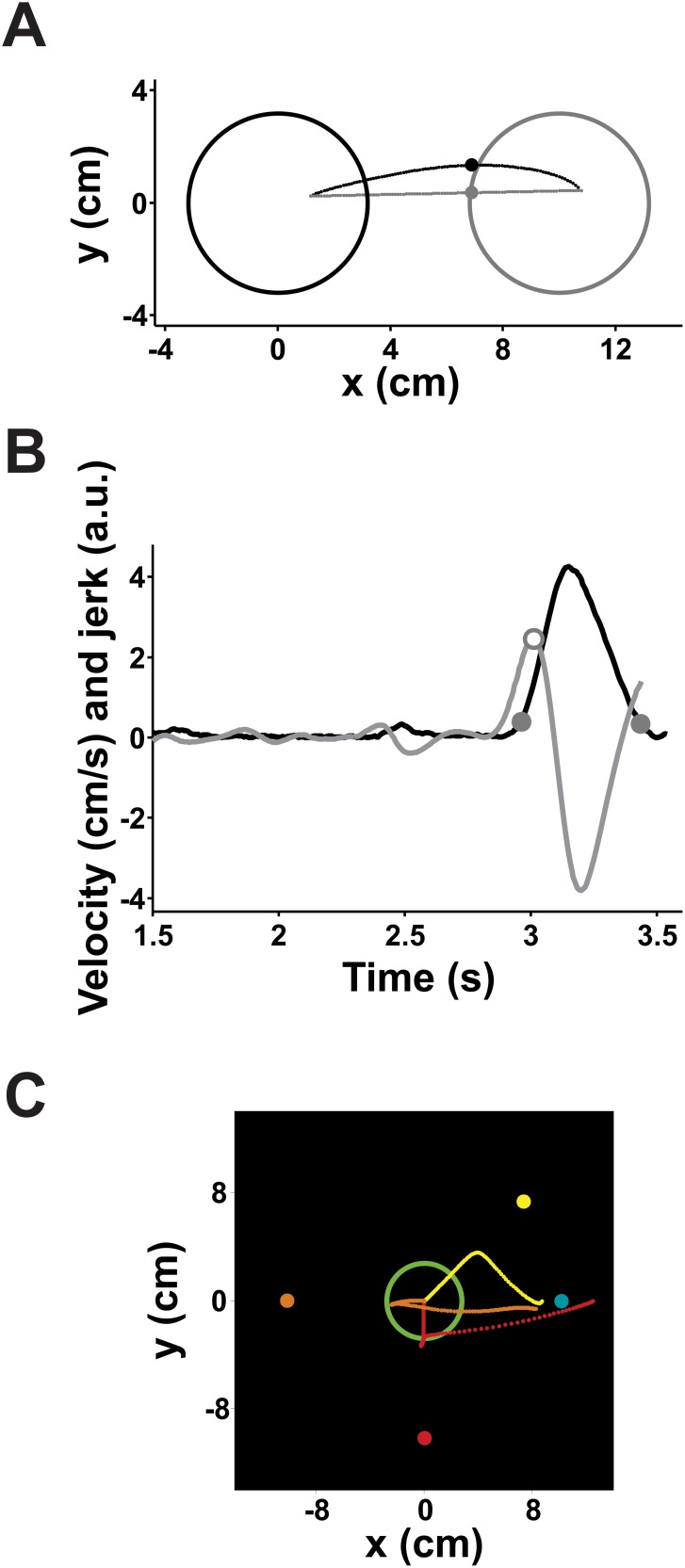
Example of a trajectory to a response circle and its velocity and jerk profile. **A)** Real (black dotted line) and straight (gray dotted line) interpolated trajectories from the central circle (left black-outlined circle) to the response circle (right gray-outlined circle). Both trajectories share a common beginning and ending point and are composed of 100 equidistant points. The enlarged point in each trajectory signals the place in which the largest distance between both trajectories lies, which is considered as the maximum angle of the trajectory. **B)** Velocity (black line) and jerk (gray line) of the movement depicted in A). The two filled circles in the velocity trace signal the beginning and the end of the movement and the open circle in the jerk trace signals the presence of one peak, implying that this particular trajectory had no submovements. The jerk has been multiplied by an arbitrary factor for illustration purposes. **C)** Three trajectories that were considered to have changed, each with a different angle between targets. The original target’s position is depicted in the same color as the trajectory.

We first defined the beginning and the end of the movement towards the target as the moment in which velocity went above and below 10% of the maximum velocity reached during the trial, respectively (Fig. 12B). Then, we defined the RT as the interval between the appearance of the response circles and movement initiation, whereas MT comprised the interval between movement beginning and end. The Maximum Velocity was defined as the highest velocity value reached during MT.

#### Number of submovements

The third derivative of the position, known as *jerk*, has long been considered as an important kinematic variable and it has been argued that one of the goals of the motor system is to minimize it during movement [59]. To determine if the trajectory was composed of more than one movement, we counted the number of jerk peaks within the movement (Fig. 12B). The rationale behind our approach was that if two or more peaks were found, this would imply that the subject had decelerated and reaccelerated during the movement, thus making a sub-movement.

#### Measures of trajectory curvature

The projected trajectory was interpolated to obtain 100 evenly distributed points. A straight line that joined the starting and ending points of this trajectory was also interpolated with the same *x* coordinates as the interpolated original trajectory (Fig. 12A). We then obtained the movement area by integrating the area between these two curves. We also calculated the absolute perpendicular distance between each corresponding point of both interpolations. The maximum distance was converted to degrees in polar coordinates and was used to find decision changes as described below.

#### Decision changes

Finally, we calculated the angle at the trajectory point in which the maximum deviation from the straight line had occurred (Fig. 12A). This angle was then used to find decision changes: any trajectory with an angle higher than half the angle between the targets was considered a decision change. However, when the angle between both targets was 180°, this algorithm was no longer useful. In this case, we considered that if the first 5 trajectory samples were negative, a decision change had occurred. Typical examples of trajectories considered to have a decision change are depicted in Figure 12C.

#### Independent variables

The six movement variables were subjected to Repeated Measures ANOVAs with 3 independent variables: 1) the position of the chosen response circle which determined movement direction; 2) the angle subtended by the two response circles, and 3) the categorized interval. The level of statistical significance to reject the null hypothesis was α = 0.05 and we corrected for sphericity with the Greenhouse-Geisser method.

### Model

We used a mutual inhibition network model [31] to mimic our most critical results. We used a simplified network model of discharge rate neurons. The model is composed of two mutually inhibiting neural populations, 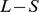, that represented the time passage, temporary memory storage, decision making, and movement execution; and 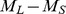 that only accumulated the passage of time and its memory trace. Additionally, two readout units, *Large* and *Short* average the activity of 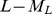 and 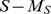, respectively (Fig. 6A). The 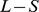 population is instantiated by the following equations:
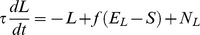


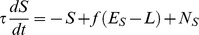
whereas the memory population by:



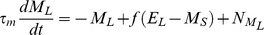


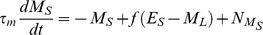
where 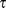 (155 ms) is a constant for time passage and was estimated by minimizing the difference between the interval duration of subjective equality (p = 0.5) in the simulation and the mean of the presented intervals (687.5 ms). 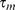 (500 ms) is a time constant for memory storage, 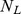, 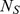, 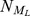 and 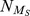 are white noise, and 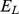 and 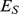 are the excitatory inputs to the 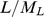 and 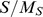 nodes, respectively, which vary as a function of the task phase as depicted in Figure 4B–F. The output function *f* is a commonly used sigmoidal function (Fig. 6B) given by:



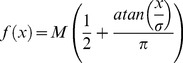
where M is the maximum response of the node and 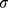 is the slope of the sigmoidal function. Thus, this function represents how the output of each node changes with its input. For 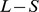, Figure 6B shows the activity of *L* as function of *S* and for different values of 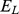. It can be observed that as *S* increases its inhibitory output to *L*, *L*’s output decreases. Also, as the task phase changes, 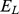 changes as well, shifting the curve. The corresponding curve for *S* as a function of *L* can be plotted with an axes rotation as shown in Figures 6C–F (first and third row panels), where it can be noticed that 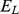 and 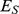 change for the stimulus, delay and reaction phases and determines interval quantification, memory storage, reaction and movement time. Analogously, memory populations, 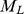 and 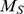 are mutually inhibited as shown in Figure 6C–F (second and forth row panels). Finally, the downstream reading areas (DRA), one for each category, integrate the output of their corresponding nodes: DRA_L_ is the mean of the *L* and 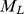
’s output, whereas DRA_S_ does the same for *S* and 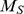. We performed 10,000 simulations for every interval in order to estimate the model’s categorical performance, as well as the RT and MT for correct and incorrect trials. It is important to remark that only 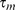 and the thresholds associated with the definition of RT and MT were adjusted, using the least-square method with respect to the subjects’ RT and MT (Table 3).

**Table 3:** Threshold values for movement onset as a function of the angle between the targets and for the end of movement.

Angle	Threshold
45°	0.61
90°	0.59
135°	0.56
180°	0.54
Movement	0.04

We also tried to replicate our behavioral results by developing a random-walk model. In this case, evidence is accumulated in a node over time (as a diffusion process) until a lower 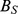 or upper 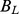 bound is reached, which triggers the corresponding decision process. During the presentation of the interval (I), the node accumulates information over time with a mean drift 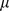 and standard deviation 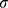. In our task, both the 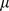 and standard deviation 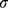 are the same across the categorized intervals. Therefore, the mean response at time *I* is equal to 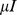 and standard deviation is equal to 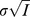. The information accumulation stops during the delay period, namely 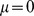, and the node simply maintains the same mean response that was reached after information acquisition. Finally, after target onset, the node reaches one of the bounds, 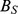 or 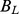. In our model 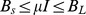, which implies that both bounds and the trajectory of the node are always positive, since time accumulation is always positive. From Palmer et al. equation A6 [60], the probability of stopping at the lower bound 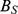 depends on the difference between the mean response and each bound, with 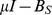 and 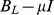 for the short and long bounds, respectively. When the drift rate is 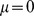, the diffusion model allows for the estimation of the probability of correct decisions as follows:
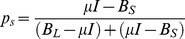
where, 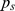 is the probability of correct decisions for a short interval (s). For our time categorization task this probability can be rewritten as the following linear function:



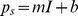
where 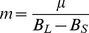 and 
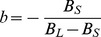
. Therefore, this random-walk model predicts a linear psychometric function and cannot account for the sigmoidal profile of the subjects’ categorical performance observed here (Figures 2A and 8A). Diffusion models have been successfully applied to explain the response times and the accuracy of subjects’ performance in two-alternative forced choice tasks [60]. However, in visual movement-direction discrimination tasks, the psychometric functions are calculated using Palmer et. al., equation A13 [60] as a function of 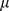 that is proportional to the stimulus strength. Thus, under these conditions a diffusion model produces a sigmoidal shape in the psychometric function.

## Supporting Information

Dataset S1 Page 1: Mean reaction times (RT) as a function of the categorized intervals for each of the twenty subjects for correct and incorrect trials. Page 2: Mean movement times (MT) as a function of the categorized intervals for each of the twenty subjects for correct and incorrect trials.
(PDF)

Dataset S2 Mean reaction (RT) and Movement times (MT) as a function of the angle between the two response targets for each of the 20 subjects.
(PDF)
