# Supplementary material for: Linking Perception, Cognition, and Action: Psychophysical Observations and Neural Network Modelling
Source: PLoS One. 2014 Jul 16;9(7):e102553. doi: 10.1371/journal.pone.0102553 (PMC4100910; doi:10.1371/journal.pone.0102553)
Supplement: Dataset S1 — Page 1: Mean reaction times (RT) as a function of the categorized intervals for each of the twenty subjects for correct and incorrect trials. Page 2: Mean movement times (MT) as a function of the categorized intervals for each of the twenty subjects for correct and incorrect trials. (PDF) [file pone.0102553.s001.pdf]

correct trials RT (ms)

Interval (ms)

Subject	450	500	619	669	709	756	870	920
**1**	488.627	507.882	557.431	503.429	522.393	547.179	511.380	527.200
**2**	477.681	488.114	480.732	510.953	510.026	507.370	484.545	489.014
**3**	472.889	481.600	483.208	552.000	583.796	538.848	510.414	503.986
**4**	552.887	544.234	616.238	664.000	595.281	607.288	584.286	566.153
**5**	552.875	578.125	570.969	559.245	596.320	539.156	554.967	575.138
**6**	519.250	522.638	547.963	583.961	625.903	534.512	567.723	591.738
**7**	453.571	460.333	502.000	525.582	502.045	487.207	490.400	488.300
**8**	573.899	554.894	677.940	709.925	625.196	628.760	588.645	563.242
**9**	468.708	482.507	503.327	498.053	489.057	474.873	484.145	475.083
**10**	476.556	490.806	506.246	567.605	562.327	564.153	525.592	516.814
**11**	534.657	563.516	521.436	527.920	578.412	547.600	530.985	520.727
**12**	517.479	583.268	575.328	683.964	625.364	533.824	571.854	489.309
**13**	563.697	605.667	588.476	609.566	587.667	599.757	589.102	575.817
**14**	528.861	532.060	524.776	572.000	550.085	557.119	552.746	557.556
**15**	594.485	625.130	632.600	705.514	638.525	593.630	564.814	578.548
**16**	514.130	530.358	601.021	621.286	546.089	519.393	511.455	516.343
**17**	516.783	503.453	600.479	560.278	556.739	585.333	551.241	549.191
**18**	531.912	578.594	574.444	619.875	615.245	586.917	554.302	576.985
**19**	494.803	499.486	536.739	535.532	637.429	652.167	582.125	524.631
**20**	572.172	604.339	663.600	824.852	682.840	630.914	709.700	668.292

incorrect trials RT (ms)

Subject	450	500	619	669	709	756	870	920
**1**	707.400	567.500	580.071	546.826	541.500	530.000	557.955	512.917
**2**	447.000	561.500	500.000	515.517	509.176	513.444	464.833	437.000
**3**	NaN	549.000	618.895	554.308	540.870	639.833	623.500	679.000
**4**	662.000	669.125	629.471	618.760	620.800	590.833	747.500	NaN
**5**	NaN	NaN	636.625	590.842	660.298	600.889	599.000	548.714
**6**	NaN	612.000	707.056	583.381	609.244	589.000	617.000	688.143
**7**	549.000	736.000	523.167	515.353	489.700	508.233	513.364	553.000
**8**	659.333	679.500	659.591	752.219	718.462	759.500	814.600	900.833
**9**	NaN	609.000	494.400	481.824	486.486	520.765	559.000	NaN
**10**	NaN	NaN	588.933	558.765	598.261	601.692	739.000	726.500
**11**	585.500	617.375	549.000	555.979	569.857	699.588	512.333	558.167
**12**	1063.000	561.000	656.091	547.412	653.282	705.974	714.792	732.118
**13**	548.667	619.333	594.667	626.053	666.000	658.857	796.538	737.083
**14**	NaN	633.800	595.929	563.568	573.923	510.800	1098.000	NaN
**15**	902.500	813.000	645.824	615.400	661.938	726.154	777.846	911.200
**16**	685.000	517.400	599.583	586.270	713.630	799.438	557.333	650.000
**17**	827.000	824.750	607.000	595.139	666.115	548.222	689.214	920.000
**18**	651.750	579.750	596.778	591.050	588.000	552.000	774.222	690.333
**19**	479.000	NaN	679.333	698.900	587.121	658.833	763.938	622.000
**20**	850.500	1029.400	861.432	756.578	791.182	914.500	734.583	1056.000

correct trials MT (ms)

Interval (ms)

Subject		450	500	619	669	709	756	870	920
	**1**	516.090	487.926	516.552	519.184	493.786	515.536	532.640	517.817
	**2**	621.464	616.300	615.857	614.512	605.211	609.907	609.848	630.028
	**3**	466.292	488.214	497.962	522.152	535.082	483.788	490.714	501.662
	**4**	623.831	617.156	672.905	687.636	647.298	670.273	620.700	626.958
	**5**	594.097	559.472	572.828	591.566	577.760	588.178	573.754	570.646
	**6**	326.458	342.609	335.519	358.431	343.161	350.279	332.800	345.923
	**7**	448.271	468.203	437.485	452.509	447.864	450.414	444.260	438.467
	**8**	308.246	301.318	319.500	286.100	329.652	324.240	334.403	339.758
	**9**	422.375	448.380	462.827	433.658	464.429	434.600	441.942	443.250
	**10**	465.653	468.722	450.088	415.895	458.041	478.288	459.479	480.900
	**11**	639.829	646.953	619.333	652.240	711.118	673.873	651.970	653.742
	**12**	500.155	528.366	560.770	554.873	521.879	534.824	572.958	529.782
	**13**	603.924	646.939	607.302	639.547	589.407	609.027	682.102	632.467
	**14**	378.722	389.925	363.017	314.679	384.678	377.045	381.831	392.472
	**15**	509.727	506.043	503.673	500.108	496.725	512.935	506.458	494.242
	**16**	389.754	383.851	415.396	415.857	412.378	386.286	410.682	420.179
	**17**	434.580	452.500	481.000	433.111	455.674	454.074	434.000	443.088
	**18**	376.691	357.641	382.267	408.125	392.306	398.500	380.937	384.985
	**19**	435.493	435.306	464.609	471.129	512.071	493.633	454.804	456.215
	**20**	420.875	420.339	403.400	408.000	429.740	447.914	436.400	415.846

incorrect trials MT (ms)

Subject									
	**1**	510.800	494.000	540.714	529.913	500.659	556.500	549.545	488.417
	**2**	527.333	625.500	562.063	612.517	601.676	680.722	640.333	838.000
	**3**	NaN	560.500	497.263	497.718	505.000	410.167	374.500	608.000
	**4**	826.000	660.875	637.529	654.640	661.533	652.833	711.000	NaN
	**5**	NaN	NaN	544.250	552.316	566.340	601.852	630.091	574.857
	**6**	NaN	320.667	365.944	324.286	336.171	331.862	444.000	346.571
	**7**	501.500	352.000	485.667	448.529	472.720	489.791	495.636	494.500
	**8**	340.000	317.833	364.091	332.750	322.654	322.727	306.200	329.333
	**9**	NaN	478.000	457.850	460.853	443.027	422.706	487.667	NaN
	**10**	NaN	NaN	487.267	502.912	445.435	421.154	448.000	433.500
	**11**	522.000	650.500	645.576	629.319	660.476	678.118	660.667	692.167
	**12**	472.000	784.000	495.364	640.588	583.128	614.211	542.250	568.176
	**13**	692.333	694.333	773.444	640.895	629.778	633.200	644.462	647.000
	**14**	NaN	357.400	351.929	374.091	321.308	351.400	254.000	NaN
	**15**	474.333	507.667	508.176	493.971	506.656	472.231	518.385	509.200
	**16**	440.667	382.600	383.250	393.081	378.296	362.375	414.833	449.400
	**17**	503.667	520.000	455.625	445.139	460.923	454.333	427.214	411.500
	**18**	351.000	411.375	384.148	366.475	407.087	431.083	348.222	353.833
	**19**	454.000	NaN	576.333	490.500	465.759	480.643	507.250	520.000
	**20**	387.625	454.000	422.108	440.200	461.818	504.429	430.500	445.857
