# Supplementary material for: Linking Perception, Cognition, and Action: Psychophysical Observations and Neural Network Modelling
Source: PLoS One. 2014 Jul 16;9(7):e102553. doi: 10.1371/journal.pone.0102553 (PMC4100910; doi:10.1371/journal.pone.0102553)
Supplement: Dataset S2 — Mean reaction (RT) and Movement times (MT) as a function of the angle between the two response targets for each of the 20 subjects. (PDF) [file pone.0102553.s002.pdf]

Subject	RT (ms) Angle (degrees)			MT (ms) Angle (degrees)		
	45	90	180	45	90	180
1	517.844	536.208	526.693	521.292	516.193	513.938
2	485.823	497.557	499.396	626.146	617.000	604.917
3	503.177	519.146	539.906	501.005	491.755	491.099
4	563.354	612.245	598.974	650.052	642.573	631.302
5	560.781	586.120	582.411	583.625	569.099	580.323
6	558.656	572.214	580.495	342.531	341.141	340.599
7	471.708	484.281	523.276	472.495	461.406	435.974
8	636.359	627.193	642.490	339.318	308.255	317.115
9	479.922	487.266	487.813	447.854	450.630	433.302
10	525.932	515.339	552.552	471.599	458.188	461.495
11	527.667	546.906	574.885	664.500	649.167	648.859
12	577.516	583.828	630.672	566.182	532.083	548.328
13	590.557	616.625	617.859	634.365	649.740	619.307
14	559.839	545.135	544.880	360.776	380.375	383.573
15	633.516	640.339	627.016	507.406	505.276	494.464
16	548.708	550.943	582.594	407.885	403.271	388.198
17	567.510	554.214	591.792	466.495	446.781	434.792
18	571.438	588.703	587.297	395.635	384.042	370.458
19	559.536	575.260	554.411	468.677	462.396	457.865
20	688.922	724.875	699.609	424.943	429.901	433.641
